# Initiation to heroin injecting among heroin users in Sydney, Australia: cross sectional survey

**DOI:** 10.1186/1477-7517-2-2

**Published:** 2005-02-15

**Authors:** Carolyn A Day, Joanne Ross, Paul Dietze, Kate Dolan

**Affiliations:** 1 National Drug and Alcohol Research Centre, University of New South Wales, SYDNEY NSW 2052, Australia; 2 Turning Point Alcohol and Drug Centre Inc. & Deakin University School of Health and Social Development, MELBOURNE VIC Australia

**Keywords:** heroin, injecting, initiation, risk behaviour

## Abstract

**Background:**

Heroin injection is associated with health and social problems including hepatitis C virus (HCV) transmission. Few studies have examined the circumstances surrounding initiation to heroin injecting, especially current users initiating others. The current study aimed to examine the age of first heroin use and injection; administration route of first heroin use; relationship to initiator; the initiation of others among a group of heroin users; and to examine these factors in relation to HCV status and risk.

**Method:**

Heroin users in Sydney were recruited through needle and syringe programs, a methadone clinic and snowballing. Participants were interviewed about their own initiation to heroin use, blood-borne virus risk and knowledge, and whether they had initiated others to heroin injecting. Information on HCV status was collected via self-report. Data was analysed using univariate and multivariate statistical techniques for Normally distributed continuous and categorical data.

**Results:**

The study recruited 399 heroin users, with a mean age of 31 years, 63% were male, 77% reported heroin as their primary drug and 59% were HCV positive (self-report). Mean age at first heroin use and injection was 19 and 21 years, respectively. The majority of heroin users commenced heroin use via injecting (65%), younger users (<25 years, 25–30 years) were less likely than older users (>30 years) to commence heroin use parenterally. Participants were initiated to injection mainly by friends (63%). Thirty-seven percent reported initiating others to heroin injection, but few factors were related to this behaviour. Those with longer heroin using careers were more likely to report initiating others to heroin injection, but were no more likely to have done so in the preceding 12 months. Participants who had initiated others were more likely to have shared injecting equipment (12 vs 23%), but were no more likely to be HCV positive (self-report) than those who did not.

**Conclusion:**

Interventions to prevent heroin users initiating others to injecting are necessary. Peer groups may be well positioned to implement such interventions.

## Background

Heroin is one of the most commonly injected illicit substances worldwide [1,2]. Heroin injection is associated with a range of harms including poor health [3,4], poor psychosocial functioning [5] and increased risk of fatal and non-fatal heroin overdose [6,7]. Heroin injection is also a significant risk factor for blood-borne viral infections (BBVI) such as hepatitis C, hepatitis B and HIV [8-12]. Despite these harms little is known about initiation to heroin injecting.

Hepatitis C is probably the most prevalent health infection among injecting drug users (IDU) worldwide. The prevention of hepatitis C has proven difficult; unlike hepatitis B there is currently no vaccine available and programs which have been successful in reducing HIV have had only a small impact on the HCV epidemic [13,14]. It has therefore increasingly been acknowledged that prevention of initiation to drug injection is likely to be one of the most effective prevention strategies for blood-borne viral transmission [15]. Heroin injecting in particular is associated with increased HCV prevalence compared to injection of amphetamine – the two most commonly injected drugs in Australia [16].

A decrease in the age of initiation to drug use, including heroin use, across birth cohorts has been documented in both Australia [17,18] and the United States [19]. This decrease in the age of heroin initiation has been associated with greater poly drug use, unintentional overdose and criminal behaviour, independent of years of heroin use [17]. Better understanding of the circumstances surrounding initiation to injecting heroin use is needed if appropriate interventions are to be formulated.

Crofts *et al*. examined a number of factors surrounding initiation to injecting among a group of young, recently initiated IDUs [20]. They found that the majority of their sample had instigated the first injecting episode, but were assisted or injected by friends; only a very small proportion were injected by a family member. The research also found that more females than males reported being injected by their partner, a pattern consistent with the literature in that females are more likely than males to have an IDU sex partner and are less likely to be able to inject themselves [21-23]. A recent Canadian study of street youth also found that females were more likely than males to be injected by their partner or lover [24]. However, research from the United States found only 13% of IDU interviewed were initiated by their sex partner, with no difference between males and females; indeed females were more likely to be initiated by another female [25].

Little attention has been paid to the initiation of others into injecting [15]. One small study of a brief intervention to prevent initiation to injecting found that 40% of the 86 IDU interviewed had initiated a mean of two people to injecting [26]. Crofts *et al*. found that 47% of recently initiated IDUs had also initiated another into injecting and of those who had, few informed new initiates of BBVI risk [20]. The relationship between initiators' BBVI status and risk behaviour was not reported, so it was not possible to determine whether those engaging in risky behaviours were more or less likely to initiate others to injecting. Moreover, the study focused on young or recently initiated injectors, so the role of age and experience in initiating others to injecting was unable to be determined.

Crofts and colleagues found that the majority of recently initiated IDU were aware of HIV and hepatitis B and that the viruses could be transmitted via shared injecting equipment [20]. A much smaller proportion knew of HCV, though those who were aware of the virus were also aware that it could be transmitted via shared injecting equipment [20]. However, information on serostatus or hepatitis B vaccination status was not reported, nor was injecting risk behaviours such as needle and syringe sharing.

This study examined initiation to heroin injection. Specifically the study aimed to examine: 1) age of first heroin use and first heroin injection; 2) route of first heroin use; 3) relationship to initiator; and 4) the initiation of others among a group of heroin users. These factors were then examined in relation to demographic variables such as gender, ethnicity, level of education and also blood borne virus status.

## Methods

### Procedure

Heroin users in Sydney were volunteers recruited through needle and syringe programs and a methadone clinic. In order to sample a range of heroin users, snowballing, facilitated by a peer interviewer, was also used. Drug users were eligible to participate if they reported the use of heroin at least once a month in the preceding six months. All participants gave informed consent to be interviewed and received AUD20 for travel expenses.

### Measures

A structured questionnaire was administered to participants by trained interviewers. Information was sought on blood-borne virus status and knowledge, and initiation to heroin use and included questions on age of first heroin use, age of first heroin injection, relationship to initiator and the number of times participants had initiated others to injecting.

Information on HCV status was collected via self-report. Self-reported HCV status has a concordance of approximately 80% for those who have been tested [27,28]. For the purposes of this study, self-reported HCV is relevant as risk behaviour is influenced by the belief about one's status, not actual status [29].

To determine ethnicity, participants were asked how they identified ethnically, those identified as being of Aboriginal or Torres Strait Islander decent were categorised as Indigenous, those not born in Australia and those born in Australia but who identified as belonging to another ethnic group were categorised as 'other Australian'.

The study was approved by four institutional ethics committees: University of New South Wales Human Research Ethics Committee, Central Sydney Area Health Service Ethics Review Committee, South Western Sydney Area Health Service Research Ethics Committee, and South Eastern Sydney Area Health Service Research Ethics Committee.

### Analysis

Continuous variables were assessed using *t*-tests and one-way analysis of variance. Linear regression was employed to test the relationship between continuous variables. The chi square (χ^2^) statistic was used for univariate analysis of categorical data. Multiple logistic regression, using backward elimination, was used for multivariate analysis to examine independent relationships between dichotomous variables. All data were analysed using SPSS version 11.01.

## Results

### Sample characteristics

The sample consisted of 399 heroin users, of whom 63% were male. The mean age of participants was 31 years (SD 8.2, range 17, 58). The majority of participants were born in Australia (78%), a small proportion of which identified as another ethnic group. Participants were categorised as either 'Australian' (67%), Indigenous (17%) or 'other-Australian' (16%). The majority (81%) of participants had attended secondary school, but only 42 (11%) participants had tertiary education. Thirty-four (9%) participants had completed primary (elementary) school only. Sixty-one percent of participants had a history of incarceration.

Heroin was the primary drug used by 77% of the sample, with a median of 9.5 (range <1, 39) years of use and injecting. The majority (98%) of the sample injected heroin; 14% of the sample had been injecting heroin for three years or less and 80% for more than three years (18 cases had missing data for this variable). Those who did not inject heroin were excluded from analysis pertaining to injecting.

### Age of initiation to heroin use and injection

The mean age at first heroin use was 19 years, (SD 6.0, range 9–43 years) and the mean age of first injection was 21 years (SD 6.3, range 13–43 years). The mean age of first heroin use and first heroin injection was similar for males (18 years for both) and females (18 and 19 years, respectively). There were no differences in terms of ethnicity for either age of first heroin use (18 years for all groups) or first heroin injection (18, 19 and 18 years for Australians, 'other Australians' and Indigenous Australians respectively).

For those who completed primary school only, the mean age of first heroin use was 17 (SD 5.4) years and 19 (SD 6.0) years for those who attended secondary school or above, the difference failed to reach significance (*t*_395 _= -1.89, p = .0.56). Participants who completed primary school only, were a mean age of 17 (SD 4.8) years when they first injected heroin and the mean age of first injection for those who attended secondary school and above was 20 (SD 6.3) years (*t*_386 _= -2.46, p = .014).

Linear regression was used to examine the relationship between current age and age of initiation to heroin use and injecting. There was a significant relationship between participants' current age and age of first heroin use (β = 0.41, p < .001). Similarly, current age and age of first injection were also significantly related (β = 0.39, p < .001).

### Route of first heroin administration

Heroin was most commonly first administered by injection (65%). For the 141 participants who used non-parenteral routes of administration on initiation of heroin use, the most common method was smoking (burning and chasing), with 28% of the sample reporting initiating heroin use with this method. Only seven percent first used heroin intranasally, orally or by other means.

Participants who first injected heroin and those who first used heroin non-parenterally, commenced heroin use at similar ages (19 and 20 years, respectively). Those who initiated heroin use by injection had been using heroin for more years than those who initiated heroin use non-parenterally (13 years vs 9 years respectively, *t*_379 _= 4.36, p = 0.001).

Route of administration was also associated with ethnicity: 74% of Indigenous participants commenced heroin use via injection, 69% of 'Australians' and 36% of 'other Australians' (χ^2 ^= 26.40, p < .001). There were no differences in the proportion of participants who commenced heroin use via injecting in terms of gender (males 64% and females 65%) or level of education (primary education 77%, secondary education 64%).

Logistic regression was used to determine the factors independently associated with initiation to heroin use via injecting. Variables entered into the model were age, gender, ethnicity and age at first injection (before or after 18 years). Level of education was not entered into the model as there were too few participants with only primary school level of education who commenced heroin use via a non-parenteral route of administration.

The final model was significant (χ^2 ^= 43.92, 4*df*, p < .001) and the Hosmer and Lemeshow test indicated good fit (χ^2^= 1.5 6*df*, p = .96). Gender was not a characteristic independently associated with route of first heroin injection and was removed from the model. Participants aged less than 25 years and 25–30 years were less likely than those aged 30 years or more to initiate heroin use via injecting. 'Other Australians' were also less likely to reported initiating less injecting (Table 1).

**Table 1:** Characteristics of those who first injected heroin, using multivariate logistic regression

**Characteristic**	**No. participants**	**%1^st ^injected**	**Adjusted odds ratio**	**95% CI**	**P**
Age					
31+	184	77	-	-	
25–30	106	60	0.48	0.28–0.83	.008
≤ 24	96	54	0.34	0.19–0.61	<.001
Ethnicity					
Australian	269	67	-	-	-
ATSI	69	74	1.18	0.61–2.26	ns
Other Australians	61	36	0.30	0.16–0.55	<.001
Age 1st injected					
≥ 18	164	77	-	-	
<18	234	56	0.63	0.39–1.02	.059

CI = Confidence interval

### Relationship to initiator

Participants were usually taught to inject by a friend (63%), family member (14%) or their partner (11%). Ten percent of the sample reported 'other', which was typically self-taught. Males and females differed significantly in terms of who taught them to inject (χ^2 ^= 24.75, *df *= 2, p < .001; Table 2). Specifically, a greater proportion of females were taught to inject by their partner than were males (χ^2 ^= 23.96, *df *= 1, p < .001), while more males were taught to inject by a friend (or other) than females (χ^2 ^= 11.77, *df *= 1, p < .001). The relationship between participants and their 'initiators' did not differ according age of first injection, ethnicity or level of education across those initiated by friends, family or their partner.

### Initiating others

Over a third (37%) of participants reported having taught someone to inject drugs and 17% had done so in the preceding 12 months. Among those who had ever taught someone to inject (n = 149), the median numbers of people taught was three (range: 1–200) and two (range: 1–50) in the preceding 12 months.

Similar proportions of males and females reported teaching someone to inject ever (38% vs 37%) and in the preceding 12 months (16% vs 19%). There was also no significant difference in terms of ethnicity or mean age of those who had ever taught someone to inject ever or in the preceding 12 months. Those who had taught someone else to inject heroin had been injecting for a greater mean number of years than the remainder of the sample (13 v. 10 years; *t *= -3.08, *df *= 370, p = 0.002). However, this difference diminished for those who had recently (preceding 12 months) taught someone to inject (11 years for both groups).

### Blood-borne viral infections

Ninety-two percent of participants reported being tested for HCV infection; 62% in the six months preceding interview and 78% in the 12 months preceding interview. The median number of weeks since the last test was 19 (range: 1–572 weeks). Two-hundred and thirty-five participants (59% of the total sample) self-reported being HCV positive.

Participants who reported being HCV positive initiated heroin injecting at a younger mean age (19 years, SD 5.7) than the remainder of the sample (21 years, SD 6.8). HCV positive participants were more likely to report initiating heroin use via injecting than those who were HCV negative (72% vs 58%, χ^2 ^= 9.14, *df *= 1, p = 0.003). IDUs typically become infected with HCV early in their injecting career [30], thus analysis was stratified by years of injecting (≤ 3 years vs >3 years). The relationship remained only for those who had been injecting for more than three years (61% vs 76%, χ^2 ^= 7.93, *df *= 1, p = 0.005). There was no difference in self reported HCV status between those who had initiated someone to injecting in the preceding 12 months and those who had not.

Eighty-eight percent of participants had reported being tested for HIV a median of six months prior to interview (range <1–144). Of these, seven (2% of the total sample) reported being HIV positive. One-hundred and forty-seven participants (37%) reported having been vaccinated against hepatitis B. There were no differences between those who reported being vaccinated against hepatitis B and those who had not in terms of age at first heroin injection, initial route of administration and initiating some one else to injecting in the preceding 12 months.

Only a small number of participants (9%) reported having used a needle or syringe after someone else in the month preceding interview. Sharing needles and syringes was not associated with mean age at initiation to heroin injection or initial route of heroin administration. More participants who had recently initiated someone to injecting (i.e. preceding 12 months) reported sharing needles and syringes (17%) than those who had not recently initiated someone (8%), though the difference failed to reach significance (χ^2 ^= 3.51, *df *= 1, p = .061).

Sharing (borrowing or lending) injection paraphrenia in the month preceding interview was reported by just over half (52%) the sample. Participants who had engaged in this behaviour were more likely to have initiated heroin use via injecting (71% vs 61%, χ^2 ^= 4.37, *df *= 1, p < .037) and to have recently initiated someone to injecting (12% vs 23%, χ^2 ^= 7.95, *df *= 1, p < .005) compared to those who not shared injection paraphernalia. There was no difference in terms of age at first heroin injection.

## Disscussion

This study has identified factors associated with initiation to heroin injecting. The majority of heroin users commenced heroin use via injecting, though those who initiated heroin use via this method had been using heroin for a longer period than those who initiated heroin use via non-parenteral methods. Participants were initiated to injection by a range of people, mainly friends. A large proportion of study participants also reported initiating another person to heroin injection, a practice that was associated with longer heroin use careers and recent sharing of injecting equipment.

Increasing attention has been paid to the role of IDUs in initiation to injecting drug use [15], though few studies have examined the prevalence of this behaviour. Crofts and colleagues postulate that the behaviour can be understood as communicable and modelled as in the case of infectious diseases [20]. Fewer participants in the current study reported initiating others to injecting than that reported by Crofts *et al*. (37% vs 47%) and only 17% reported doing so in the preceding 12 months. Crofts *et al*. sampled only young injecting drug users (i.e. 17–24 years) [20], whereas the injecting experience of participants sampled for this study ranged from less than one year to 39 years and younger participants were no more likely than older participants to report initiating others to injecting.

A third of the sample initially used heroin by non-parenteral routes of administration. Participants from ethnically diverse backgrounds (i.e. born outside Australia or identify other than Australian) were more likely than other participants to have first used heroin by means other than injecting, which is consistent with other Australian research which found smoking heroin to be more common among Indochinese than Caucasian heroin users [31].

Participants who commenced heroin use via injecting were also older than those who first used a non-injecting route of heroin administration, possibly indicating an overall shift toward non-injecting routes of administration. A number of interventions aimed at reducing the incidence of transition to injecting have received attention [15]. One study found that brief interventions delivered through existing drug services can have an impact on injecting drug users' behaviour, however, the study was modest and further research is warranted [26].

The current study found a direct significant relationship between current age and age of first heroin use and first heroin injection. This result is consistent with other Australian research [17,18]. Nevertheless the finding is subject to bias due to 'right censoring' of the data [17]. For example, an 18 year old recruited into the study cannot have an age of initiation above 18 years, while a 30 year old can have any age of initiation up to 30 years, and be included in the study even if they commenced injecting at 25 years [for a more detailed discussion see [17]]. There are numerous explanations for this decrease in initiation to heroin use; the rapid expansion of the Australian heroin market between 1996 and 2000, where the price of heroin decreased concomitant with an increase in purity and availability from 1996 to 2000 [32], may in part explain this phenomenon, though greater examination of the structural determinants of drug use are also warranted [33].

Although the proportion of participants reporting using a needle or syringe after another person (sharing) in the preceding month was low, it is consistent with other research examining transitions to injecting [31], but lower than Australian national estimates of this behaviour [16]. It is possible that the data is subject to a social desirability bias and thus under-reported. Participants reported comparatively high levels of 'indirect' sharing (sharing injecting paraphernalia other than needles and syringes), a phenomenon recently found to significantly and independently increase the risk of HCV transmission [34]. This behaviour was reported more often by those who reported teaching someone to inject in the 12 months preceding interview. That those who are more likely to engage in this behaviour are also teaching others to inject is cause for concern, as it has the potential to perpetuate the problem of injecting related risk taking behaviour among new recruits.

The majority of the sample believed themselves to be HCV positive and, not surprisingly, those who injected at a younger age were more likely to be HCV positive and to have first used heroin via injection. Though importantly, there was no difference in terms of HCV status between those who had recently taught another to inject and those who had not.

## Conclusions

This study has confirmed that initiation to heroin use in Australia typically occurs via injection, though this is less apparent among younger heroin uses. The study has also found that more than a third of heroin users have initiated others into injecting, with close to a fifth having done so recently. Many of those who engaged in this behaviour tended to take greater injection related risks which has important implications for the transmission of blood-borne infections. A better understanding of the circumstances surrounding the initiation to heroin injection is needed. Peer-led interventions, which have been found to be effective in changing IDUs' attitudes and behaviours [26], may have a role to play in reducing the number of heroin users initiating others to injecting.

## Competing interests

The author(s) declare that they have no competing interests.

## Authors' contribution

C Day coordinated the study, was responsible for the statistical analysis and writing the paper. P Dietze was responsible for study design at the national level, secured funding and provided comments on the manuscript. J Ross and K Dolan supervised all aspects of the work and provided extensive comments on the manuscript.
